# Omega-3 Fatty Acids and Inflammatory Processes

**DOI:** 10.3390/nu2030355

**Published:** 2010-03-18

**Authors:** Philip C. Calder

**Affiliations:** Institute of Human Nutrition, School of Medicine, University of Southampton, MP887 Southampton General Hospital, Tremona Road, Southampton SO16 6YD, UK; Email: pcc@soton.ac.uk

**Keywords:** leukocyte, neutrophil, macrophage, monocyte, eicosanoid, cytokine, interleukin, fish oil

## Abstract

Long chain fatty acids influence inflammation through a variety of mechanisms; many of these are mediated by, or at least associated with, changes in fatty acid composition of cell membranes. Changes in these compositions can modify membrane fluidity, cell signaling leading to altered gene expression, and the pattern of lipid mediator production. Cell involved in the inflammatory response are typically rich in the n-6 fatty acid arachidonic acid, but the contents of arachidonic acid and of the n-3 fatty acids eicosapentaenoic acid (EPA) and docosahexaenoic acid (DHA) can be altered through oral administration of EPA and DHA. Eicosanoids produced from arachidonic acid have roles in inflammation. EPA also gives rise to eicosanoids and these often have differing properties from those of arachidonic acid-derived eicosanoids. EPA and DHA give rise to newly discovered resolvins which are anti-inflammatory and inflammation resolving. Increased membrane content of EPA and DHA (and decreased arachidonic acid content) results in a changed pattern of production of eicosanoids and resolvins. Changing the fatty acid composition of cells involved in the inflammatory response also affects production of peptide mediators of inflammation (adhesion molecules, cytokines *etc.*). Thus, the fatty acid composition of cells involved in the inflammatory response influences their function; the contents of arachidonic acid, EPA and DHA appear to be especially important. The anti-inflammatory effects of marine n-3 PUFAs suggest that they may be useful as therapeutic agents in disorders with an inflammatory component.

## 1. Introduction

Inflammation is a normal defense mechanism that protects the host from infection and other insults; it initiates pathogen killing as well as tissue repair processes and helps to restore homeostasis at infected or damaged sites. It is typified by redness, swelling, heat, pain and loss of function, and involves interactions amongst many cell types and the production of, and responses to, a number of chemical mediators. Where an inflammatory response does occur, it is normally well regulated in order that it does not cause excessive damage to the host, is self-limiting and resolves rapidly. This self-regulation involves the activation of negative feedback mechanisms such as the secretion of anti-inflammatory mediators, inhibition of pro-inflammatory signaling cascades, shedding of receptors for inflammatory mediators, and activation of regulatory cells. As such, when controlled properly, regulated inflammatory responses are essential to remain healthy and maintain homeostasis. Pathological inflammation involves a loss of tolerance and/or of regulatory processes [1]. Where this becomes excessive, irreparable damage to host tissues and disease can occur. Irrespective of the cause of the inflammation, the response involves four major events:

- An increased blood supply to the site of inflammation;
- Increased capillary permeability caused by retraction of endothelial cells. This permits larger molecules, not normally capable of traversing the endothelium, to do so and thus delivers soluble mediators to the site of inflammation;
- Leukocyte migration from the capillaries into the surrounding tissue. This is promoted by release of chemoattractants from the site of inflammation and by the upregulation of adhesion molecules on the endothelium. Once in the tissue the leukocytes move to the site of inflammation;
- Release of mediators from leukocytes at the site of inflammation. These may include lipid mediators (e.g., prostaglandins (PGs), leukotrienes (LTs)), peptide mediators (e.g., cytokines), reactive oxygen species (e.g., superoxide), amino acid derivatives (e.g., histamine), and enzymes (e.g., matrix proteases) depending upon the cell type involved, the nature of the inflammatory stimulus, the anatomical site involved, and the stage during the inflammatory response. These mediators normally would play a role in host defense, but when produced inappropriately or in an unregulated fashion they can cause damage to host tissues, leading to disease. Several of these mediators may act to amplify the inflammatory process acting, for example, as chemoattractants. Some of the inflammatory mediators may escape the inflammatory site into the circulation and from there they can exert systemic effects. For example, the cytokine interleukin (IL)-6 induces hepatic synthesis of the acute phase protein C-reactive protein, while the cytokine tumour necrosis factor (TNF)-α elicits metabolic effects within skeletal muscle, adipose tissue and bone.

## 2. Fatty Acid Composition of Cells Involved in Inflammation and its Modification by Marine n-3 Fatty Acids

**Figure 1:**
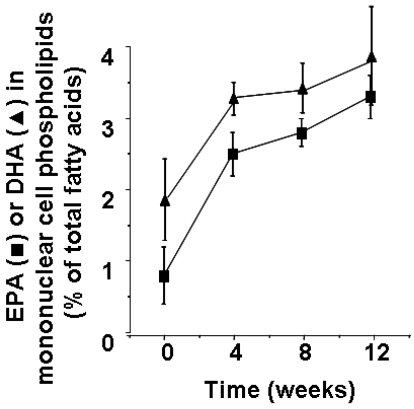
Time course of incorporation of EPA and DHA into human blood mononuclear cells. Healthy subjects supplemented their diet with fish oil capsules providing 2.1 g EPA plus 1.1 g DHA per day for a period of 12 weeks. Blood mononuclear cell phospholipids were isolated at 0, 4, 8 and 12 weeks and their fatty acid composition determined by gas chromatography. Data are mean ± SEM from 8 subjects and are from Yaqoob *et al.* [19].

Polyunsaturated fatty acids (PUFAs) are important constituents of the phospholipids of all cell membranes. Laboratory animals that have been maintained on standard chow have a high content of arachidonic acid (20:4n-6) and low contents of eicosapentaenoic acid (20:5n-3; EPA) and docosahexaenoic acid (22:6n-3; DHA) in the bulk phospholipids of tissue lymphocytes [2,3], peritoneal macrophages [4,5,6,7,8], alveolar macrophages [9,10], Kupffer cells [10], and alveolar neutrophils [11,12,13]. Feeding laboratory animals a diet containing fish oil, which provides EPA and DHA, results in a higher content of these fatty acids in lymphocytes [3], macrophages [4,5,6,7,9,10], Kupffer cells [10] and neutrophils [11,12,13]; typically enrichment in marine n-3 PUFAs is accompanied by a decrease in content of arachidonic acid. The bulk phospholipids of blood cells representing those that become involved in inflammatory responses (e.g., neutrophils, lymphocytes, monocytes) and collected from humans consuming typical Western diets contain about 10 to 20% of fatty acids as arachidonic acid, with about 0.5 to 1% EPA and about 2 to 4% DHA [14,15,16,17,18,19,20,21,22,23,24,25], although there are differences between the different phospholipid classes in terms of the content of these fatty acids [16]. The fatty acid composition of these cells can be modified by increasing intake of marine n-3 fatty acids [14,15,16,17,18,19,20,21,23,24,25,21,23]. This occurs in a dose response fashion [25] and over a period of days to weeks, with a new steady-state composition reached within about 4 weeks (Figure 1). Typically the increase in content of n-3 PUFAs occurs at the expense of n-6 PUFAs, especially arachidonic acid. 

## 3. Mechanisms by which Polyunsaturated Fatty Acids can Influence Inflammatory Cell Function

PUFAs can influence inflammatory cell function, and so inflammatory processes, by a variety of mechanisms as follows:

- PUFA intake can influence complex lipid, lipoprotein, metabolite and hormone concentrations that in turn influence inflammation;
- Non-esterified PUFAs can act directly on inflammatory cells via surface or intracellular “fatty acid receptors” – the latter may include transcription factors like peroxisome proliferator activated receptors (PPARs);
- PUFAs can be oxidized (enzymatically or non-enzymatically) and the oxidized derivatives can act directly on inflammatory cells via surface or intracellular receptors – oxidation can occur to the non-esterified form of the PUFA or to PUFAs esterified into more complex lipids including circulating or cell membrane phospholipids and intact lipoproteins such as low density lipoprotein (LDL);
- PUFAs can be incorporated into the phospholipids of inflammatory cell membranes(as described above). Here they play important roles assuring the correct environment for membrane protein function, maintaining membrane order (“fluidity”) and influencing lipid raft formation [26]. Membrane phospholipids are substrates for the generation of second messengers like diacylglycerol and it has been demonstrated that the fatty acid composition of such second messengers, which is determined by that of the precursor phospholipid, can influence their activity [27]. In addition, membrane phospholipids are substrates for the release of (non-esterfied) PUFAs intracellularly – the released PUFAs can act as signaling molecules, ligands (or precursors of ligands) for transcription factors, or precursors for biosynthesis of lipid mediators which are involved in regulation of many cell and tissue responses, including aspects of inflammation and immunity (see below). Thus, changes in membrane phospholipid fatty acid composition, as described above, can influence the function of cells involved in inflammation via:
  ○ alterations in the physical properties of the membrane such as membrane order and raft structure;
  ○ effects on cell signaling pathways, either through modifying the expression, activity or avidity of membrane receptors or modifying intracellular signal transduction mechanisms that lead to altered transcription factor activity and changes in gene expression;
  ○ alterations in the pattern of lipid mediators produced, with the different mediators having different biological activities and potencies (see below).

The multitude of potential mechanisms involved and their complexity has made it difficult to fully understand the actions of PUFAs within inflammatory processes. This difficulty has been further compounded by the variety of experimental approaches that have been used, including the method of presentation of PUFAs of interest to inflammatory cells in order to study their effects. For example, many *in vitro* studies have exposed cells to non-esterified fatty acids, often at concentrations that might not be achieved physiologically. Thus, effects of non-esterified PUFAs on responses of lymphocytes [2], monocytes [28], macrophages [8,29,30,31,32,33], neutrophils [34,35,36] and endothelial cells [37,38,39] have been demonstrated. These effects may involve a direct effect of the non-esterified PUFA or of an oxidized derivative of the PUFA [40,41,42] or they may be secondary to incorporation of the PUFA into cell membrane phospholipids. Physiologically, the concentration of non-esterified n-3 PUFAs (and also arachidonic acid) is quite low. These fatty acids are carried in the bloodstream at much higher concentrations in more complex lipids (triglycerides, phospholipds, cholesteryl esters) within lipoproteins. Many of the cell types involved in inflammatory responses express lipoprotein receptors (e.g., LDL receptor, very low density lipoprotein receptor, scavenger receptors) and so are able to take up intact lipoproteins, subsequently utilising the fatty acid components. Thus, lipoproteins may affect inflammatory cell function [43,44], perhaps due to their component fatty acids. Inflammatory cells may also access fatty acids from lipoproteins by hydrolysing them extracellularly as has been demonstrated for macrophages [45] and lymphocytes [46]. Thus, cells involved in inflammatory processes are exposed to fatty acids, including PUFAs, in many different forms, and they may access fatty acids from their environment by a variety of mechanisms. The effect of the form of presentation of PUFAs to inflammatory cells can be examined in the cell culture setting and studies to date indicate that non-esterified fatty acids [28,29,30,31,32,33,34,35,36,37,38,39], complex lipids like triglycerides [46], intact lipoproteins [44], and oxidized forms of fatty acids and other lipids [40,41,42] all influence inflammatory cell responses, frequently with different effects or different potencies of n-6 and n-3 PUFAs. 

Following increased dietary intake of marine n-3 PUFAs their concentrations increase in complex lipids within the bloodstream (triglycerides, phospholipids, cholesteryl esters), as well as within the membrane phospholipids of cells and tissues including those involved in inflammatory responses (see above), and there is a small increase in their concentration within the circulating non-esterfied fatty acid pool; the latter increase is small because circulating non-esterfied fatty acids derive principally from adipose tissue triglyceride breakdown and adipose tissue triglycerides contain very little EPA and DHA. Thus, following increased intake of EPA and DHA, both the cells involved in inflammation and their extracellular environment (e.g., blood plasma) are enriched in those fatty acids, so that the n-3 PUFA enriched inflammatory cells will be in contact with n-3 PUFA-rich complex lipids and lipoproteins. Many studies have examined the effect of increased intake of marine n-3 PUFAs on the function of cells typically involved in inflammation taken from the bloodstream (neutrophils, eosinophils, monocytes, lymphocytes) or, in the case of animal studies tissues and subsequently cultured. In many, probably most, cases the *in vivo* situation is not maintained during the *ex vivo* culture period, in that the n-3 PUFA enriched cells are maintained in an environment that is different from that to which they were exposed *in vivo i.e.,* to an n-3 PUFA poor environment. Thus, the *in vivo* situation is not replicated in the *ex vivo* setting. This hampers the full interpretation of the findings of such research. 

## 4. Lipid Mediators: Biosynthesis, Roles in Inflammation, and the Impact of Marine n-3 fatty acids

### 4.1. Eicosanoids Generated from Arachidonic Acid

**Figure 2:**
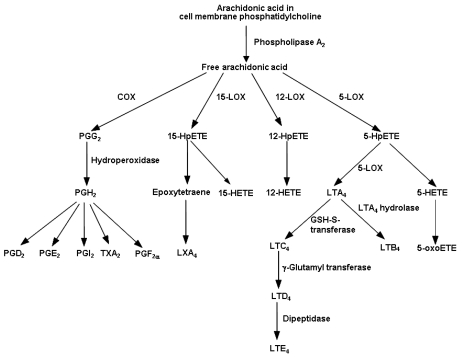
Outline of the pathway of eicosanoid biosynthesis from arachidonic acid. COX, cyclooxygenase; HETE, hydroxyeicosatetraenoic acid; HpETE, hydroperoxyeicosatetraenoic acid; LOX, lipoxygenase; LT, leukotriene; LX, lipoxin; oxoETE, oxoeicosatetraenoic acid; PG, prostaglandin; TX, thromboxane.

Eicosanoids are key mediators and regulators of inflammation and immunity and are generated from 20 carbon PUFAs. Eicosanoids, which include PGs, thromboxanes, LTs and other oxidised derivatives, are generated from arachidonic acid by the metabolic processes summarized in Figure 2. Eicosanoids are involved in modulating the intensity and duration of inflammatory responses [47,48], have cell- and stimulus-specific sources and frequently have opposing effects. Thus, the overall physiological (or pathophysiological) outcome will depend upon the cells present, the nature of the stimulus, the timing of eicosanoid generation, the concentrations of different eicosanoids generated and the sensitivity of target cells and tissues to the eicosanoids generated. Because of the relatively high amount of arachidonic acid in membrane phospholipids of cells involved in inflammation, this fatty acid is typically the major precursor for eicosanoid mediators, which are produced in greatly increased amounts upon cellular stimulation. Thus, amongst the mix of eicosanoids produced, those synthesized from arachidonic acid (e.g., PGE_2_ and LTB_4_) predominate although the exact eicosanoid profile depends upon the cell type concerned (e.g., neutrophils and mast cells produce a lot of PGD_2_ whereas monocytes produce a lot of PGE_2_) and the nature of the stimulus; the profile will also change over time as the nature of the response to the stimulus alters. In general arachidonic acid-derived eicosanoids act in a pro-inflammatory way, although this is an over-simplification since it is now recognised that PGE_2_, for example, has both pro- and anti-inflammatory effects, and that another eicosanoid derived from arachidonic acid, lipoxin A_4_, is anti-inflammatory [49,50,51,52].

### 4.2. Fatty Acid Modification of Eicosanoid Profiles

Animal studies have shown a direct relationship between arachidonic acid content of inflammatory cell phospholipids and ability of those cells to produce PGE_2_ [53], such that PGE_2_ production is increased by arachidonic acid feeding [53] and decreased by EPA or DHA feeding [53,54,55]. It is well documented that PGE_2_ and 4 series-LT production by human inflammatory cells can be significantly decreased by fish oil supplementation of the diet for a period of weeks to months [14,15,16,18,56,57]. 

**Figure 3:**
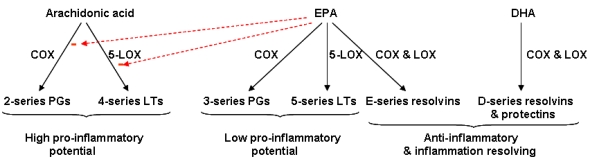
General overview of synthesis and actions of lipid mediators produced from arachidonic acid, EPA and DHA. COX, cyclooxygenase; LOX, lipoxygenase; LT, leukotriene; PG, prostaglandin.

EPA is also a substrate for the cyclooxygenase and lipoxygenase enzymes that produce eicosanoids, but the mediators produced have a different structure from the arachidonic acid-derived mediators, and this influences their potency. Increased generation of 5-series LTs has been demonstrated using macrophages from fish oil-fed mice [55] and neutrophils from humans supplemented with oral fish oil for several weeks [14,15,16]. The functional significance of the generation of eicosanoids from EPA is that EPA-derived mediators are often much less biologically active than those produced from arachidonic acid (Figure 3). For example EPA-derived LTB_5_ is 10- to 100-fold less potent as a neutrophil chemoattractant compared with LTB_4_[58,59]. Furthermore, EPA-derived eicosanoids may antagonise the action of those produced from arachidonic acid, as was recently demonstrated for PGD_3_ vs. PGD_2_[60]. However, in some cases arachidonic acid-derived and EPA-derived eicosanoids appear to behave with similar potency (e.g., inhibition of TNF-α production by monocytes [61,62]). 

### 4.3. Resolvins: Novel Anti-Inflammatory and Inflammation Resolving Mediators Produced from EPA and DHA

**Figure 4:**
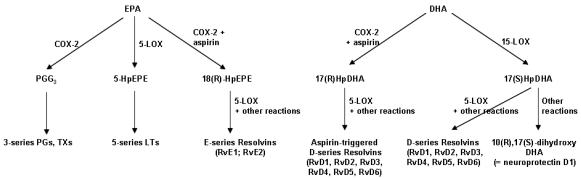
Outline of the pathway of synthesis of resolvins and related mediators from EPA and DHA. COX, cyclooxygenase; HpDHA, hydroperoxydocosahexaenoic acid; HpEPE, hydroperoxyeicosapentaenoic acid; LOX, lipoxygenase; LT, leukotriene; PG, prostaglandin; Rv, resolvin; TX, thromboxane

EPA and DHA also give rise to resolvins and related compounds (e.g., protectins) through pathways involving cyclooxygenase and lipoxygenase enzymes (Figure 4) [63,64,65]. These mediators have been demonstrated in cell culture and animal feeding studies to be anti-inflammatory and inflammation resolving (Figure 3). For example, resolvin E1, resolvin D1 and protectin D1 all inhibit transendothelial migration of neutrophils, so preventing neutrophilic infiltration at sites of inflammation, resolvin D1 inhibits IL-1β production, and protectin D1 inhibits TNF and IL-1β production (see [65] for references). The role of resolvins and related compounds may be very important because resolution of inflammation is important in shutting off the ongoing inflammatory process and in limiting tissue damage.

## 5. Influence of Marine n-3 Fatty Acids on Leukocyte Chemotaxis

A number of dietary supplementation studies with fish oil have demonstrated a time-dependent decrease in chemotaxis of human neutrophils and monocytes towards various chemoattractants including LTB_4_, bacterial peptides and human serum [14,15,16,66,67,68,16,66]. Both the distance of cell migration and the number of cells migrating were decreased. Despite the high dose of marine n-3 PUFAs used in many of these studies (3.1 to 14.4 g EPA+DHA/day), a dose response study by Schmidt *et al.* [69] suggests that near-maximum inhibition of chemotaxis occurs at an intake of 1.3 g EPA+DHA/day. Some studies report no effect of n-3 PUFAs on neutrophil chemotaxis [20,70,71]. An explanation for this lack of effect with regard to one of these studies [70] may be that the dose of EPA+DHA used was too low to be active (0.55 g EPA+DHA/day). However the other two studies [20,71] used higher doses of EPA+DHA, but in both cases this was a DHA-rich preparation with little EPA being provided. If this is the explanation for the lack of effect, then this suggests that the anti-chemotactic effects of fish oil might be due to EPA rather than DHA. There have been no studies attempting to discriminate the effects of EPA and DHA on chemotaxis. The mechanism by which n-3 PUFAs inhibit chemotaxis is not clear but may relate to reduced expression or antagonism of receptors for chemoattractants.

## 6. Influence of Marine n-3 Fatty Acids on Adhesion Molecules and Adhesive Interactions

Cell culture [28,37,38,39] and animal feeding studies [72,73] report decreased expression of some adhesion molecules on the surface of monocytes [28], macrophages [72], lymphocytes [73] or endothelial cells [37,38,39] following exposure to marine n-3 PUFAs. In some cases this was shown to result in decreased adhesion between leukocytes and endothelial cells. Supplementing the diet of healthy humans with fish oil providing about 1.5 g EPA+DHA/day resulted in a lower level of expression of ICAM-1 on the surface of blood monocytes stimulated *ex vivo* with interferon-γ [74]. Dietary fish oil providing 1.1 g EPA+DHA/day was found to decrease circulating levels of soluble VCAM-1 in elderly subjects [75], but it is not clear if this represents decreased surface expression of VCAM-1.

## 7. Influence of Marine n-3 Fatty Acids on Inflammatory Cytokines

### 7.1. Transcription Factors Involved in Regulating Inflammatory Gene Expression

In addition to effects on inflammation mediated by changes in the pattern of eicosanoids and other lipid mediators produced, marine n-3 PUFAs have also been shown to alter the production of inflammatory proteins including chemokines, cytokines, growth factors and matrix proteases. This effect may be mediated by altered activation of key transcription factors involved in regulating inflammatory gene expression. Two transcription factors that are likely to play a role in inflammation are nuclear factor κ B (NFκB) and PPAR-γ. NFκB is the principal transcription factor involved in upregulation of inflammatory cytokine, adhesion molecule and cyclooxygenase-2 genes [76,77]. NFκB is activated as a result of a signalling cascade triggered by extracellular inflammatory stimuli and involving phosphorylation of an inhibitory subunit (inhibitory subunit of NFκB (IκB)) which then allows translocation of the remaining NFκB dimer to the nucleus [78]. The second transcription factor, PPAR-γ, is believed to act in an anti-inflammatory manner. While PPAR-γ directly regulates inflammatory gene expression, it also interferes with the activation of NFκB creating an intriguing interaction between these two transcription factors [79]. Both NFκB and PPAR-γ may be regulated by n-3 PUFAs.

### 7.2. Fatty Acid Modulation of Inflammatory Cytokine Production and of Transcription Factor Activation

EPA and DHA inhibited endotoxin-stimulated production of IL-6 and IL-8 by cultured human endothelial cells [38,80] and EPA or fish oil inhibited endotoxin-induced TNF-α production by cultured monocytes [29,30]. EPA or fish oil decreased endotoxin-induced activation of NFκB in human monocytes and this was associated with decreased IκB phosphorylation [31], perhaps due to decreased activation of mitogen-activated protein kinases [32]. These observations suggest effects of marine n-3 PUFAs on inflammatory gene expression via inhibition of activation of the transcription factor NFκB.

Animal feeding studies with fish oil support the observations made in cell culture with respect to the effects of marine n-3 PUFAs on NFκB activation and inflammatory cytokine production. Compared with feeding corn oil, fish oil lowered NFκB activation in endotoxin-activated murine spleen lymphocytes [81]. Feeding fish oil to mice decreased *ex vivo* production of TNF-α, IL-1β and IL-6 by endotoxin-stimulated macrophages [54,82,83]. Several studies in healthy human volunteers involving supplementation of the diet with fish oil have demonstrated decreased production of TNF-α, IL-1β, IL-6 and various growth factors by endotoxin-stimulated monocytes or mononuclear cells (a mixture of lymphocytes and monocytes) [15,18,56,84,85,86], although not all studies confirm this effect. Some of the studies that fail to show an effect of n-3 PUFAs on cytokine production have provided < 2 g EPA+DHA/day [70,87,88,89,90], which may be an insufficient dose, although others have provided higher doses [19,24,91,92,93,94]. It is not clear what the reason for these discrepancies in the literature is, but technical factors are likely to be contributing factors, as discussed in detail elsewhere [95]. The relative contributions of EPA and DHA might also be important in determining the effect of fish oil. One other factor that has recently been identified is polymorphisms in genes affecting cytokine production [96]. In this study it was found that the effect of dietary fish oil upon cytokine production by human mononuclear cells was dependent upon the nature of the -308 TNF-α and the +252 TNF-β polymorphisms. 

## 8. Anti-Inflammatory Effects of Marine n-3 Fatty Acids Suggest a Therapeutic Value

Inflammation is an overt or covert component of numerous human conditions and diseases (Table 1) [1]. Although the inflammation may afflict different body compartments, one common characteristic of these conditions and diseases is excessive or inappropriate production of inflammatory mediators including eicosanoids and cytokines [1]. The foregoing discussion has highlighted that marine n-3 PUFAs can act in a number of ways to reduce inflammation. They:

- decrease production of eicosanoid mediators from arachidonic acid, many of which have pro-inflammatory roles;
- increase production of weakly inflammatory or anti-inflammatory eicosanoids from EPA;
- increase production of anti-inflammatory and inflammation resolving resolvins from EPA and DHA;
- decrease chemotactic responses of leukocytes;
- decrease adhesion molecule expression on leukocytes and on endothelial cells and decrease intercellular adhesive interactions;
- decrease production of pro-inflammatory cytokines and other pro-inflammatory proteins induced via the NFκB system.

The roles marine n-3 PUFAs in shaping and regulating inflammatory processes and responses suggest that the level of exposure to these fatty acids might be important in determining the development and severity of inflammatory diseases. The recognition that marine n-3 PUFAs have anti-inflammatory actions has lead to the idea that supplementation of the diet of patients with inflammatory diseases may be of clinical benefit. Each of the diseases or conditions listed in Table 1 is a possible therapeutic target for marine n-3 PUFAs. Perhaps unsurprisingly, supplementation trials have been conducted in most of these diseases. Those conducted in patients with rheumatoid arthritis appear to be the most successful with most trials reporting several clinical benefits [97]; these benefits are supported by meta-analyses of the available data [98,99]. Studies in patients with inflammatory bowel diseases (Crohn’s disease and ulcerative colitis) provide equivocal findings with some showing some benefits and others not [100,101]. Likewise studies conducted in patients with asthma do not provide a clear picture; most studies conducted in adults do not show a clinical benefit, while there are indications of benefits of marine n-3 PUFAs in children and adolescents, although there are few studies in those groups [102]. An extension of the latter studies is recent work in pregnancy which shows an impact of marine n-3 PUFAs on the maternal and foetal immune system [103,104,105], that may reduce risk of development of allergic type diseases in infancy [104] and childhood [106]. Although this is an emerging area with few published studies at present, the possibility of an early effect of marine n-3 PUFAs on immune maturation hints at an important, novel role for these fatty acids in early development [102,107,108]. In most other inflammatory diseases and conditions there are too few studies to draw a clear conclusion of the possible efficacy of marine n-3 PUFAs as a treatment. One exception to this may be related to cardiovascular disease morbidity and mortality. There is evidence that marine n-3 PUFAs slow the progress of atherosclerosis [109,110], which has an inflammatory component [111,112]. Further, marine n-3 PUFAs decrease mortality due to cardiovascular disease [113,114]; this may be, in part, due to stabilization of atherosclerotic plaques against rupture [115], which again has an inflammatory component [112,116]. Thus, the anti-inflammatory effects of marine n-3 PUFAs may contribute to their protective actions towards atherosclerosis, plaque rupture and cardiovascular mortality. 

**Table 1:** List of diseases and conditions with an inflammatory component in which marine n-3 fatty acids might be of benefit. Note: this list is not exhaustive.

Disease/condition
Rheumatoid arthritis
Crohn’s disease
Ulcerative colitis
Lupus
Type-1 diabetes
Cystic fibrosis
Childhood asthma
Adult asthma
Allergic disease
Chronic obstructive pulmonary disease
Psoriasis
Multiple sclerosis
Atherosclerosis
Acute cardiovascular events
Obesity
Neurodegenerative diseases of ageing
Systemic inflammatory response to surgery, trauma and critical illness

The dose of marine n-3 PUFAs required to prevent or to treat different inflammatory conditions is not clear, although it is evident that the anti-inflammatory effects of these fatty acids are dose-dependent [25]. As alluded to above, studies in healthy human volunteers suggest that an intake of >2 g EPA+DHA/day is required to affect inflammatory processes. There are few dose response studies investigating the effect of marine n-3 PUFAs in patients with inflammatory conditions. Studies in rheumatoid arthritis have used 1.5 to 7 g EPA+DHA/day (average about 3.5 g/day) and have been of long duration (3 to 12 months), with effects becoming apparent after some months [97]. One study that used two doses of n-3 PUFAS [117] reported that both doses induced benefit, but that the effect was seen earlier with the higher dose. If doses of 2 g, or even more, of EPA+DHA per day are required before an anti-inflammatory effect is seen, it is possible that some studies in patients fail to show a benefit because they have used an insufficient dose, or been of insufficient duration. It is not known whether different doses of marine n-3 PUFAs are required to treat different inflammatory conditions, but this is a possibility because the precise nature of the inflammation (*i.e.,* the cells, mediators and signaling systems involved) will differ from condition to condition [1] and it may be that these different components of inflammation show different sensitivities to n-3 PUFAs. 

## 9. Conclusions

Fatty acids influence inflammation through a variety of mechanisms; many of these are mediated by, or at least associated with, changes in fatty acid composition of cell membranes. Changes in these compositions can modify membrane fluidity, cell signaling leading to altered gene expression, and the pattern of lipid mediator production. Cells involved in the inflammatory response are typically rich in the n-6 fatty acid arachidonic acid, but the contents of arachidonic acid and of the n-3 fatty acids EPA and DHA can be altered through oral administration of EPA and DHA. Eicosanoids produced from arachidonic acid have roles in inflammation. EPA also gives rise to eicosanoids and these may have differing properties from those of arachidonic acid-derived eicosanoids. EPA and DHA give rise to newly discovered resolvins which are anti-inflammatory and inflammation resolving. Increased membrane content of EPA and DHA (and decreased arachidonic acid content) results in a changed pattern of production of eicosanoids and probably also of resolvins. Changing the fatty acid composition of inflammatory cells also affects production of peptide mediators of inflammation (adhesion molecules, cytokines *etc.*). Thus, the fatty acid composition of human inflammatory cells influences their function; the contents of arachidonic acid, EPA and DHA appear to be especially important. As a result of their anti-inflammatory actions marine n-3 PUFAs have therapeutic efficacy in rheumatoid arthritis, although benefits in other inflammatory diseases and conditions have not been unequivocally demonstrated. The anti-inflammatory effects of marine n-3 PUFAs may contribute to their protective actions towards atherosclerosis, plaque rupture and cardiovascular mortality.
